# Microclimates, land cover, and socioeconomic vulnerability shape *Anopheles* hotspots in Maryland, USA

**DOI:** 10.1186/s40249-025-01407-4

**Published:** 2026-01-20

**Authors:** Chibuike Chiedozie Ibebuchi, Somtochukwu Stella Onwah, Itohan-Osa Abu

**Affiliations:** 1 Department of Mathematics, Morgan State University, Baltimore, USA; 2 Center for Urban and Coastal Climate Science Research, Morgan State University, Baltimore, USA; 3 Health and Wellness Department, Manitoba Metis Federation, Winnipeg, MB Canada; 4 University of Manitoba, Winnipeg, Canada; 5 Department of Remote Sensing, Institute of Geography and Geology, University of Würzburg, John-Skilton-Straße 4a, 97074 Würzburg, Germany

**Keywords:** *Anopheles*, Artificial intelligence, Public health, Environmental health, Social deprivation, Microclimate, Maryland, United States

## Abstract

**Background:**

*Anopheles* mosquitoes pose notable public health concerns as competent vectors of malaria and other diseases. Although malaria is no longer endemic in the United States, recent locally acquired cases in states including Maryland highlight the need to better understand *Anopheles* dynamics in the region. This study aimed to identify geographic hotspots of *Anopheles* presence in Maryland and evaluate how land cover, microclimatic conditions, and socioeconomic vulnerability shape their spatial and temporal distribution.

**Methods:**

Monthly *Anopheles* occurrence data (1999–2024) from Global Biodiversity Information Facility (GBIF) were aggregated at county and Census Block Group (CBG) scales. Counties were ranked by mean annual presence to identify hotspots. Associations with land cover, microclimatic, and socioeconomic conditions were assessed using Spearman’s rank correlation (ρ; *P* < 0.05). At the CBG scale, significantly correlated variables (|ρ| ≥ 0.25) were used to fit an Extreme Gradient Boosting model to quantify the relative importance of environmental and socioeconomic predictors. Spatial dependence was addressed through blocked cross-validation, and model interpretability was evaluated with SHapley Additive exPlanations (SHAP) values.

**Results:**

Prince George’s and Anne Arundel Counties emerged as primary hotspots of presence with highest observer effort during the analysis period. Seasonal analysis revealed an annual cycle, with peak presence from May to September, coinciding with warmer conditions favorable to vector proliferation. SHAP analysis at the CBG-scale identified habitat availability as the most influential predictor (33.1% of total model impact for low impervious surface percentage) with woody wetland emerging as the most preferred habitat; followed by humid conditions (24.6%), and low elevation (18.2%). Notably, cooler and more humid microclimates within the warm season provide optimal habitat, reflecting fine-scale environmental controls on *Anopheles* distribution. Therefore, CBG-level analysis within Prince George’s County revealed a negative correlation between Area Deprivation Index and *Anopheles* presence (ρ = –0.35), indicating fine-scale ecological drivers—such as woody wetland habitat, low impervious surface, and humid cooler microclimates—more prevalent in affluent suburban residential neighborhoods.

**Conclusions:**

This study demonstrates that fine-scale habitat characteristics and warm-season microclimates structure *Anopheles* mosquito presence in Maryland. These insights support more spatially targeted vector control and improved public health surveillance strategies.

**Graphical Abstract:**

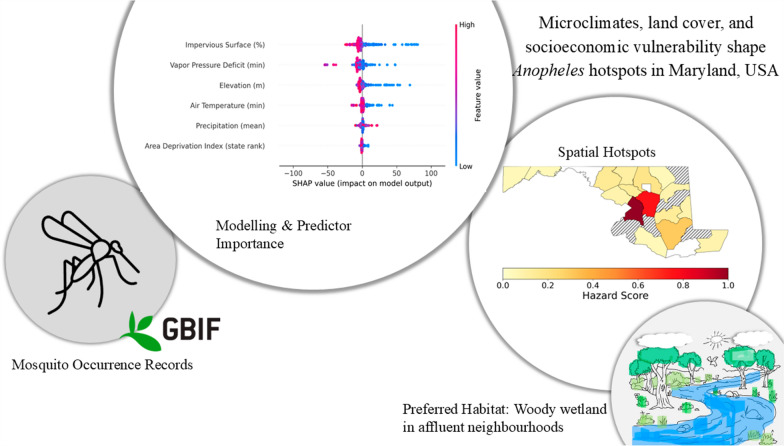

**Supplementary Information:**

The online version contains supplementary material available at 10.1186/s40249-025-01407-4.

## Background

Mosquitoes are among the most medically significant arthropods worldwide, responsible for transmitting pathogens that cause diseases such as malaria, West Nile fever, Zika disease, chikungunya, and various forms of encephalitis [1–4]. The public health burden is substantial: globally, mosquito-borne diseases cause hundreds of millions of infections and over 700,000 deaths each year [5, 6]. While malaria has historically been concentrated in tropical regions, changes in climate, land use, and human mobility have expanded the geographic ranges of several mosquito species, raising concerns even in temperate regions where these vectors were previously less predominant [7, 8]. In the United States (US), although malaria transmission is rare compared to endemic countries, locally acquired cases still occur, and arboviral diseases such as West Nile fever are persistent seasonal threats [9, 10].

*Anopheles* mosquitoes, while historically associated with malaria transmission, also contribute to the ecology of other pathogens [11]. In the US, several mosquito species are established across the Mid-Atlantic region, including Maryland, where environmental and socioeconomic conditions can contribute to localized hotspots of vector activity [12–14]. Maryland’s diverse landscape, ranging from coastal plains to urban centers, combined with its variable climate and dense human populations, creates heterogeneous habitats for mosquito breeding [14, 15]. Moreover, seasonal patterns of temperature, precipitation, and humidity influence mosquito life cycles and presence [16].

Vector surveillance in Maryland is conducted through state and local mosquito control programs, which monitor arboviral pathogen presence in mosquito pools to inform spraying and source reduction. However, routine monitoring often focuses on areas with historically high complaints or vector-borne disease activity, which may overlook other emerging at-risk communities [17–19]. Traditional entomological surveys, while essential, are costly, spatially limited, and often lack integration with socioeconomic vulnerability assessments [18, 19].

Existing research on mosquito ecology in the Mid-Atlantic has largely concentrated on climatic drivers, such as the influence of temperature and precipitation on seasonal mosquito presence [20]. Warmer temperatures can extend breeding seasons, while precipitation affects the availability of larval habitats [16]. Urban heat islands and high levels of impervious surface cover have also been linked to increased mosquito presence in some *Aedes* species, though these relationships can vary by genus and species [21]. In contrast, comparatively less attention has been paid to applying nonlinear explainable machine learning (ML) models to examine how socioeconomic factors interact with environmental conditions to shape mosquito presence at finer neighborhood scales in the Mid-Atlantic.

According to the CDC [22] and Maryland Department of Health [23], the US reports approximately 2000 imported malaria cases annually, including around 200 in Maryland. In 2023, Maryland confirmed one locally acquired case of *Plasmodium falciparum*, a parasite commonly transmitted by female *Anopheles* mosquitoes. Additionally, there is research on environmental and socioeconomic drivers of mosquito ecology in the state, particularly for *Aedes* [13, 14, 18]. However, up-to-date studies that quantify *Anopheles* risk at county or Census Block Group (CBG) scales in Maryland remain limited or absent.

Most studies of mosquito presence in the Mid-Atlantic have focused on *Culex* species, given their role in transmitting West Nile virus and other arboviruses [10, 24]. In contrast, *Anopheles* mosquitoes have received less attention in recent decades, partly because malaria has been eliminated as an endemic disease in the US [25]. However, recent reports of locally acquired malaria cases in states such as Maryland, Florida, Texas, and Arkansas indicate the need to better understand the dynamics of *Anopheles* mosquitoes, even in regions where malaria is no longer a major public health concern [26, 27]. This study provides one of the few county- and sub-county-level analyses of *Anopheles* presence in Maryland, combining long-term occurrence data with environmental and socioeconomic predictors.

We integrate occurrence records from Global Biodiversity Information Facility (GBIF) to address the following objectives: (1) identify geographic hotspots of *Anopheles* presence across Maryland at both county and CBG level; and (2) quantify how local environmental, climatic and socioeconomic factors shape mosquito presence. This multi-scale analytical framework combines a broad statewide perspective with fine-grained local analysis at the neighborhood scale, offering actionable insights for targeted vector control strategies and optimized resource allocation.

## Methods

**Fig. 1:**
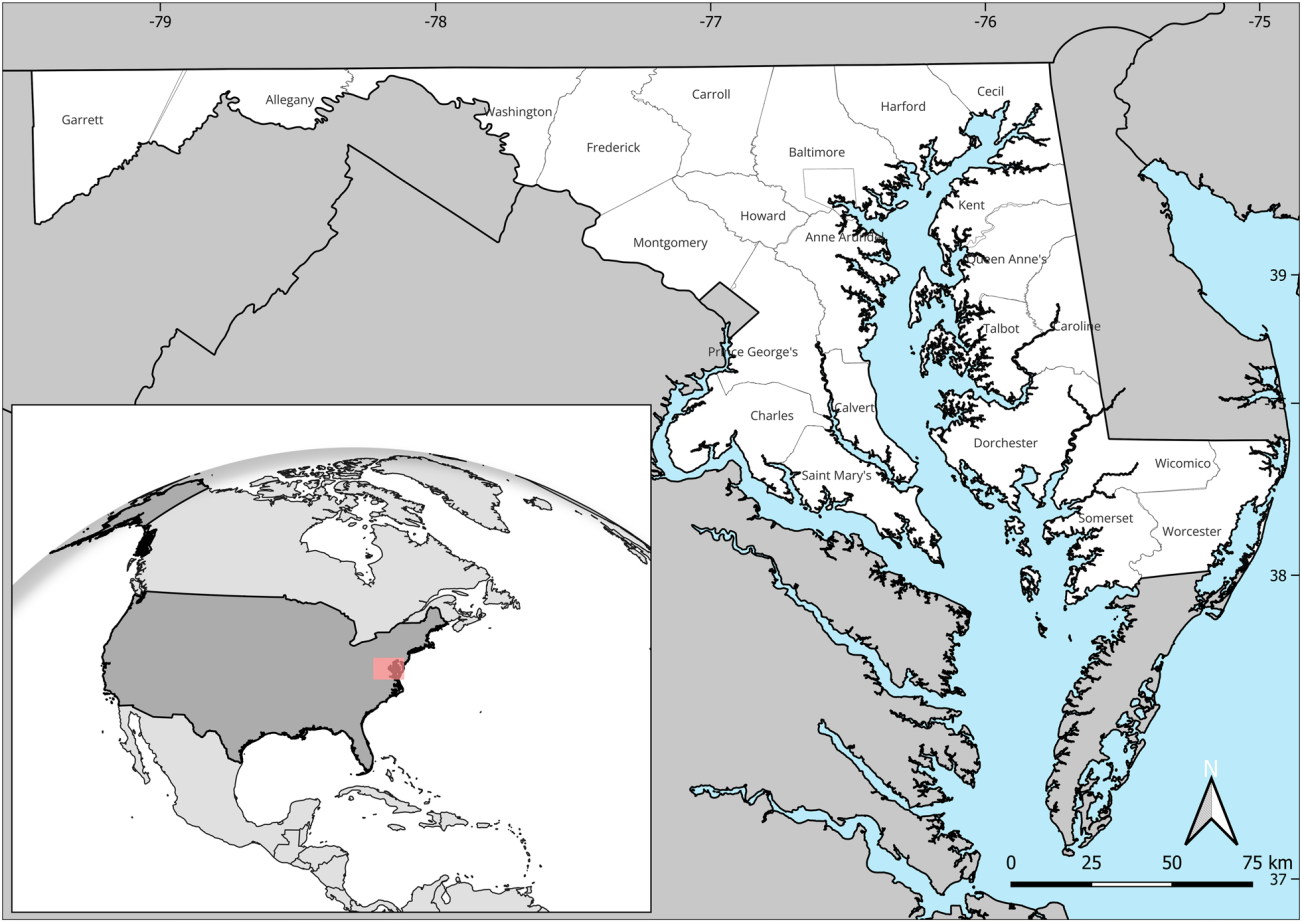
Geographical location of Maryland in the US (in red). The pinch-out image highlights the counties in Maryland. Map was created by authors using QGIS

Our analysis integrates multiple datasets capturing mosquito occurrence, environmental conditions, and socioeconomic characteristics across Maryland, US (Fig. 1). These datasets span different spatial and temporal resolutions (Table 1), enabling both county and CBG level investigations. CBGs are small U.S. Census Bureau statistical units, each containing roughly 600–3000 people, offering finer spatial resolution than counties. This granularity enables detection of neighborhood scale environmental and socioeconomic variation that may be masked in county-level averages, improving the ability to identify and analyze localized *Anopheles* hotspots.

**Table 1:** List of datasets used in this study and their relevance to mosquito spatial presence

Dataset	Source	Year/Version	Relevance to mosquito spatial presence
Mosquito occurrence point records	GBIF.org. https://www.gbif.org/	Jan 1999–Dec 2024	Provides georeferenced presence records of *Anopheles* mosquitoes from multiple sources (museum collections, academic surveys, state monitoring), used to quantify spatial and temporal patterns at county and CBG scales
CBG-level socioeconomic indicators (ADI)	University of Wisconsin School of Medicine and Public Health, Area Deprivation Index v4.0.1 [31]. https://www.neighborhoodatlas.medicine.wisc.edu/	2023	Fine-scale (CBG) composite ranking of socioeconomic disadvantage, enabling within-county mapping of areas potentially more vulnerable to mosquito-borne disease
County-level socioeconomic indicators (SVI)	CDC/ATSDR Social Vulnerability Index. https://svi.cdc.gov/dataDownloads/data-download.html	2022	Captures social and economic conditions (e.g., poverty, unemployment, housing, transportation) influencing neighborhood maintenance, exposure risk, and capacity for mosquito control at the county scale
Climate variables	PRISM Climate Group, 30-Year Normals (1991–2020) at 800 m spatial resolution https://prism.oregonstate.edu/normals/	1991–2020 climatology	Minimum/maximum air temperature, mean precipitation, and vapor pressure deficit characterize climatic conditions affecting mosquito development, survival, and activity
Elevation	USGS 10 m Digital Elevation Model, National Map Downloader. https://www.usgs.gov/the-national-map-data-delivery/gis-data-download	Latest available, as of 2025	Influences temperature, moisture retention, and hydrological patterns relevant to mosquito habitat suitability
Percent impervious surface	30 m USGS National Land Cover Database (NLCD), MRLC. https://www.usgs.gov/centers/eros/science/annual-national-land-cover-database	2024	Indicates the proportion of land covered by impermeable materials, which can influence water pooling and breeding site availability and land cover categories

*GBIF* Global Biodiversity Information Facility, *SVI* Social Vulnerability Index, *CDC* Centers for Disease Control and Prevention, *ATSDR* Agency for Toxic Substances and Disease Registry, *CBG* Census Block Group, *ADI* Area Deprivation Index, *PRISM* Parameter-elevation Regressions on Independent Slopes Model, *USGS* United States Geological Survey, *DEM* Digital Elevation Model, *NLCD* National Land Cover Database, *MRLC* Multi-Resolution Land Characteristics Consortium

### Mosquito occurrence data

We obtained *Anopheles* mosquito presence records (i.e. including *An. punctipennis, An. crucians*, and *An. quadrimaculatus*) for Maryland from GBIF [28], covering January 1999 to December 2024 – when non-zero presence records were predominantly recorded. GBIF compiles occurrence georeferenced records from multiple sources, including museum collections, academic surveys, and state monitoring programs. These *Anopheles* species were selected due to their relative presence in Maryland and competency as historical vectors of diseases such as malaria and lymphatic filariasis, as well as potential to transmit other diseases [29, 30]. Only records with valid geographic coordinates within Maryland were retained. Each record was assigned to its corresponding county and CBG using the U.S. Census TIGER/Line shapefiles 2024. The dataset was aggregated per county and per CBG to counts of presence observations, enabling characterization of trends, spatial relationships and seasonal cycles.

#### Data limitations

GBIF occurrence records are presence-only and opportunistic, and therefore subject to non-random sampling bias (e.g., spatial clustering near populated or accessible areas, variable observer effort across space and time, and reporting gaps). Absences cannot be interpreted as true absences. To reduce effort-driven artifacts in the fine-scale analysis, we limited CBG-level modeling to hotspot counties with relatively sufficient observer effort—defined as ≥ 30 unique observer-days over the study window, with coverage distributed across the warm season in multiple years. Nonetheless, residual biases, including pandemic-period disruptions to surveillance and reporting, may remain and are considered in the interpretation of result.

### Socioeconomic data

At the county level (Fig. 1), we compiled socioeconomic indicators from the Centers for Disease Control and Prevention/Agency for Toxic Substances and Disease Registry [32]. The SVI is designed to assess relative vulnerability across counties nationally, making it well-suited for inter-county comparisons. We selected measures likely to influence mosquito presence through their effects on neighborhood maintenance, exposure, and prevention capacity. These included (1) overall socioeconomic status (combining poverty, unemployment, income, and education), (2) housing and transportation vulnerability (capturing crowding, multi-unit housing, mobile homes, lack of vehicles, and group quarters); as well as individual components such as (3) the percentage of residents unemployed, or (4) without a high school diploma. Furthermore, housing-related metrics included (5) the share of crowded households, (6) proportion of multi-unit structures, and (7) percentage of mobile homes, all of which can be associated with conditions favorable for mosquito breeding, such as shared infrastructure, limited drainage, and container accumulation. We also considered (8) lack of vehicle access, which may reflect broader neighborhood disinvestment and reduced access to prevention resources.

At the CBG level, we used the 2023 Area Deprivation Index (ADI) from the University of Wisconsin School of Medicine and Public Health [31]. ADI provides a composite measure of socioeconomic disadvantage based on 17 census-derived indicators such as income, education, employment, and housing quality. We utilized ADI state decile (1 = least deprived; 10 = most deprived), which ranks CBGs within Maryland. Because ADI is reported at the CBG scale, it supports within-county analyses and fine-scale mapping of socioeconomic conditions. Higher state deciles indicate greater deprivation and, by extension, potentially greater vulnerability to environmental health risks.

### Environmental data

Environmental predictors at the CBG scale were chosen based on established associations between climate, habitat characteristics, and mosquito presence. Climate variables were obtained from the PRISM30-Year Normals, representing the 1991–2020 climatology [33]. This included minimum and maximum air temperature, mean precipitation, and minimum and maximum vapor pressure deficit, the latter serving as an indicator of atmospheric moisture and humidity conditions. CBG-level aggregation of PRISM data with 800 m resolution is aimed to capture microclimatic patterns sufficient for ecological, public health, or urban planning applications that require sub-county climate granularity.

Elevation was sourced from the U.S. Geological Survey (USGS) 10 m Digital Elevation Model through the National Map Downloader (Table 1), while percent impervious surface (and land cover categories), representing the proportion of land covered by materials such as asphalt or concrete, was obtained from the National Land Cover Database (NLCD) through the Multi-Resolution Land Characteristics Consortium [34].

In terms of data harmonization, except for SVI (county), GBIF presence data (point records) and ADI (CBG), all layers were reprojected to a common projected Coordinate Reference System for Maryland (US Contiguous Albers Equal Area, EPSG:5070), resampled as needed, and summarized to the analysis units (area-weighted means/percents within counties or CBGs).

### Methods

#### Study design and analytical framework

We employed a two-scale analytical approach. The first stage was a county-level analysis to identify *Anopheles* mosquito presence hotspots with sufficient observer efforts and evaluate associations between presence and county-level socioeconomic indicators. The second stage focused on CBG-level analysis within hotspot counties to examine fine-scale drivers using an explainable AI (XAI) framework. At both CBG and county-level, hazard score was derived by taking the mean annual presence per county/CBG, applying a log transformation to reduce skewness, and normalizing values between 0 and 1. Higher hazard scores indicate counties/CBGs with higher recorded *Anopheles* presence.

#### County-level analysis

At the county scale, total monthly *Anopheles* presence counts from January 1999 to December 2024 were aggregated and converted to mean annual values. Counties were ranked by mean annual presence to identify hotspots. For exploratory analysis, associations between mosquito presence and socioeconomic variables were evaluated using Spearman’s rank correlation (ρ), which captures monotonic relationships without assuming normality, alongside Pearson’s correlation for assessing linear associations. Statistical significance was evaluated at the 95% confidence level (*P* < 0.05).

#### CBG-level correlation analysis and variable screening

At the CBG scale, we examined environmental predictors and the ADI to assess whether socioeconomic disadvantage and habitat characteristics were associated with mosquito presence. Spearman’s ρ and *P*-values were computed, and only variables with statistically significant monotonic associations (*P* < 0.05; |ρ|≥ 0.25) were retained for XAI modeling to support a parsimonious and interpretable framework. This preliminary screening step ensured that predictors with no detectable relationship to *Anopheles* presence were excluded before model fitting, thereby reducing model complexity and computational overhead. SHAP values were then applied to the retained predictors to provide an objective, model-based ranking of their relative importance.

#### Explainable machine learning modeling and spatial blocking

To quantify the relative importance of spatial predictors, an Extreme Gradient Boosting regressor (XGBRegressor) [35] was fit to CBG-level *Anopheles* occurrence counts using the retained environmental, socioeconomic and land-cover covariates from the correlation analysis. We used gradient-boosted trees [35] over count-based models (e.g., Poisson, negative binomial) that assume specific distributional forms, to flexibly capture nonlinearities and interactions among predictors. Their non-parametric nature allows modeling of correlated covariates without strict assumptions. Spatial autocorrelation was controlled with blocked resampling. Within each county, CBG centroids were projected and partitioned into a 4 × 4 quantile grid to define spatial blocks, and GroupKFold held out entire blocks as test folds. Hyperparameters were selected with grouped five-fold cross-validation in GridSearchCV on these same blocks over a stability-oriented grid. This grid included max_depth ∈ {2,3,4}, n_estimators ∈ {400,600,800}, learning_rate ∈ {0.03,0.05,0.07}, subsample ∈ {0.8,0.9}, colsample_bytree ∈ {0.8,0.9}, and reg_lambda ∈ {0.5,1.0,2.0}, optimizing out-of-block R^2^ and reducing risk of overfitting. Models in each fold used the squared-error objective on the raw counts, and performance was summarized on the pooled out-of-block predictions across folds. For interpretability, SHAP (TreeExplainer) values were computed [36]. For each split, SHAP values were obtained only on the held-out blocks and then aggregated across folds to derive global importance (mean |SHAP|) and local effect patterns (beeswarm). This protocol limits information leakage from neighboring units, respects spatial dependence, and ensures all CBGs contribute—either to model fitting or to out-of-block explanation.

#### Data analysis

Data analysis was conducted in Python version 3.10 (Python Software Foundation, US: https://www.python.org/). Data processing and statistical computations were performed using Python packages including *pandas*, *numpy*, and *scipy*. Similaryl, XAI modeling and cross-validation were implemented using *scikit-learn*, and model interpretability was assessed using the *shap* package. Mapping and visualization were carried out using QGIS, *matplotlib* and *Cartopy*.

All statistical tests, including the non-parametric Spearman correlation, were evaluated at a significance threshold of *P* < 0.05. Because our key analytical workflow relies on distribution-free methods—including Spearman’s ρ, gradient-boosted tree models, and SHAP explanations—none of the procedures used in this study required assumptions of normality.

## Results

### County-level analysis of *Anopheles* presence

**Fig. 2:**
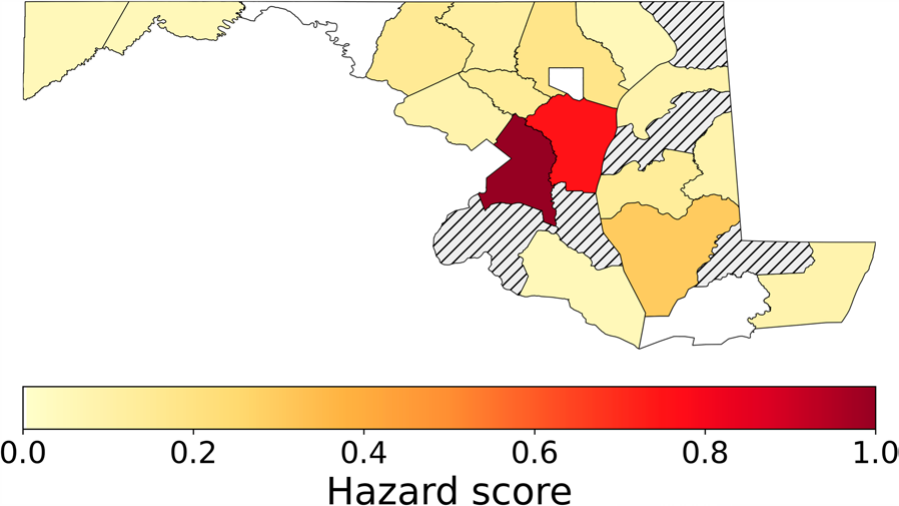
Spatial distribution of *Anopheles* mosquito presence across Maryland counties from 1999 to 2024. The striped counties indicate areas with no recorded presence during the study period

Figure 2 shows the spatial distribution of *Anopheles* mosquito presence across Maryland counties from 1999 to 2024. Two major hotspots (with sufficient observer effort) emerged—Prince George’s County (hazard score = 1.0) and Anne Arundel County (0.775)—with substantially higher *Anopheles* presence (7468 and 999, respectively) compared to other counties. Secondary clusters of elevated presence were found in Baltimore, Dorchester, and Carroll counties. Many western and lower eastern shore counties showed low or no recorded *Anopheles* presence, reflected in hazard scores near zero. This spatial pattern guided the selection of hotspot counties for subsequent fine-scale CBG-level analysis.

**Fig. 3:**
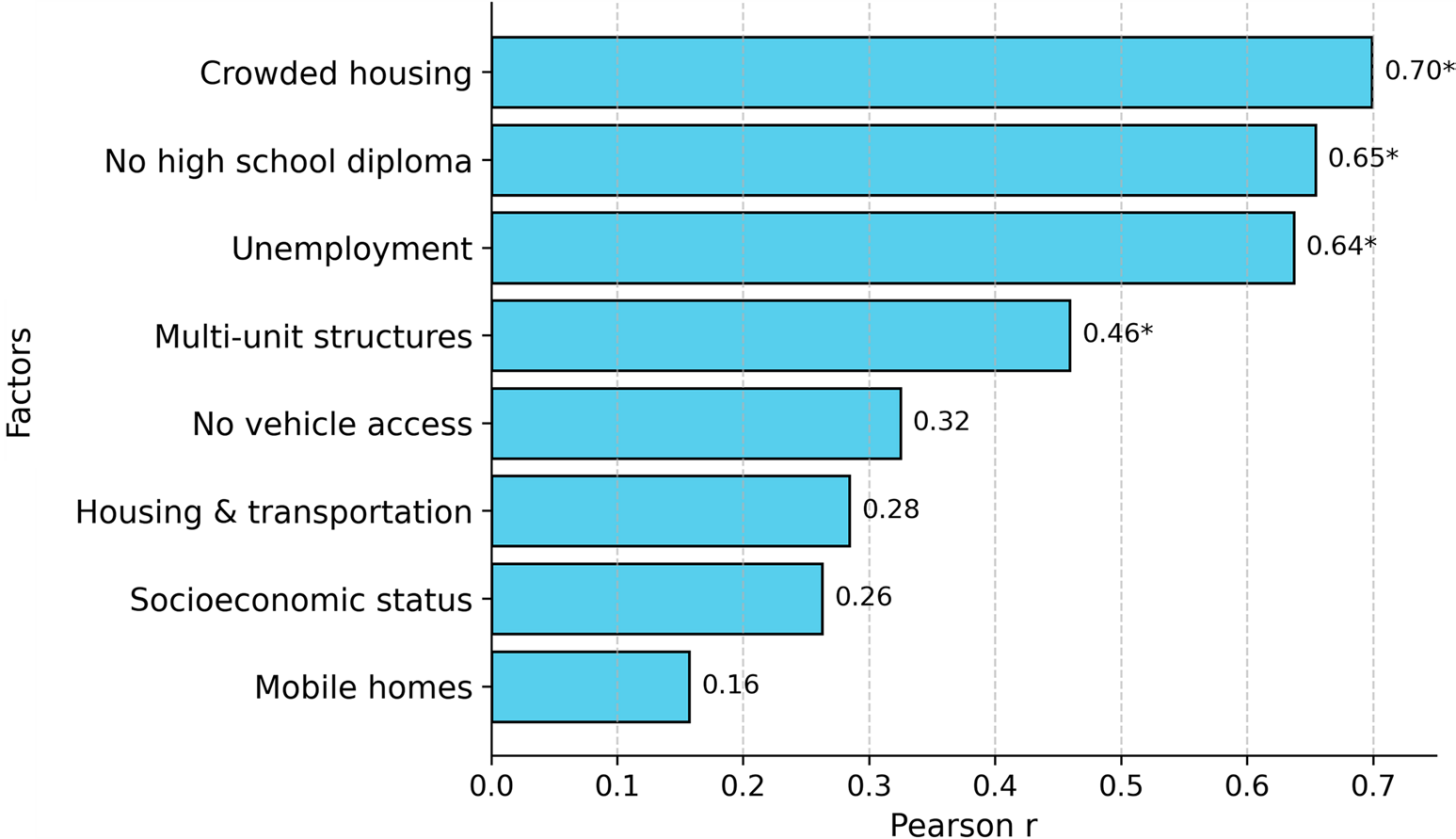
Correlation between socioeconomic factors and *Anopheles* mosquito presence count in Maryland Counties. Asterisks show significant correlations at 95% confidence level

Figure 3 shows the linear spatial relationships between county-level socioeconomic factors and *Anopheles* mosquito presence across Maryland counties. At the 95% confidence level, crowded housing (r = 0.70), no high school diploma (r = 0.65), unemployment (r = 0.64), and multi-unit structures (r = 0.46) showed statistically significant positive correlations. Other variables, including no vehicle access, housing and transportation, socioeconomic status, and mobile homes, exhibited weaker associations that were not statistically significant at a 95% confidence level. However, the correlations in Fig. 3 failed test of statistical significance at a 95% confidence level using Spearman correlations that is relatively more robust to outliers.

**Fig. 4:**
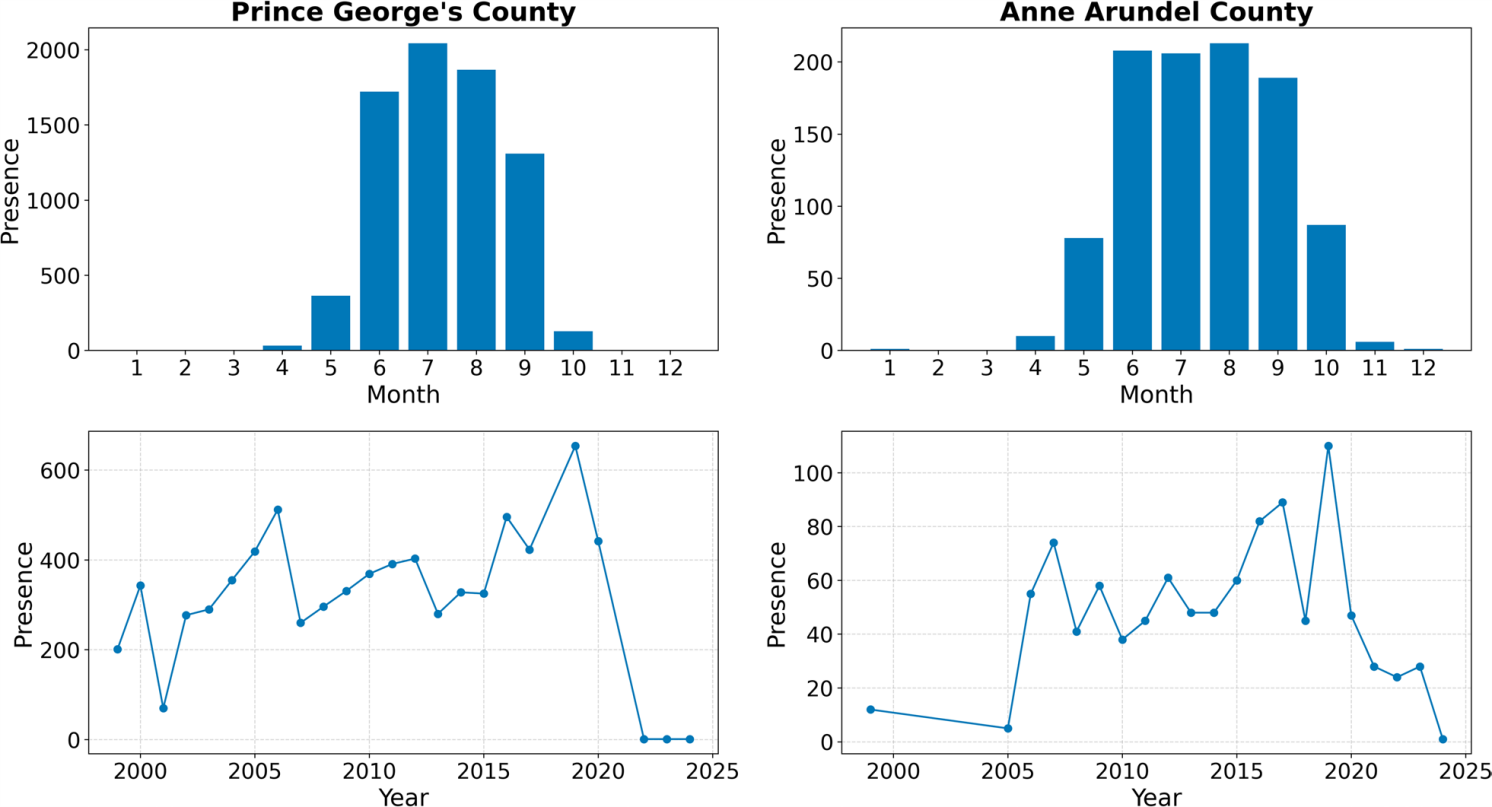
Annual cycle calculated as monthly sum of mosquito occurrence (top) and annual sum of mosquito occurrence (bottom) for the Prince George’s and Anne Arundel County (1999–2024). Zero presence was recorded in Prince George’s in 2021

Next, based on Fig. 2, we focus on the two hotspot counties—Prince George’s and Anne Arundel—for CBG-level analysis. As shown in Fig. 4, mosquito presence in both counties exhibits a clear seasonal cycle, beginning to rise in May, peaking between July and August, and declining from October onwards. At the annual scale, Prince George’s County shows an overall increasing trend in mosquito presence from 2001 to 2020, followed by a marked drop from 2021. Similarly, Anne Arundel County shows a general increase from 2004 to 2019, with a sharp decline from 2020. These sharp declines likely reflect multiple factors, including potential control measures and disruptions in monitoring and reporting.

### Neighborhood-scale level analysis of *Anopheles* presence

**Fig. 5:**
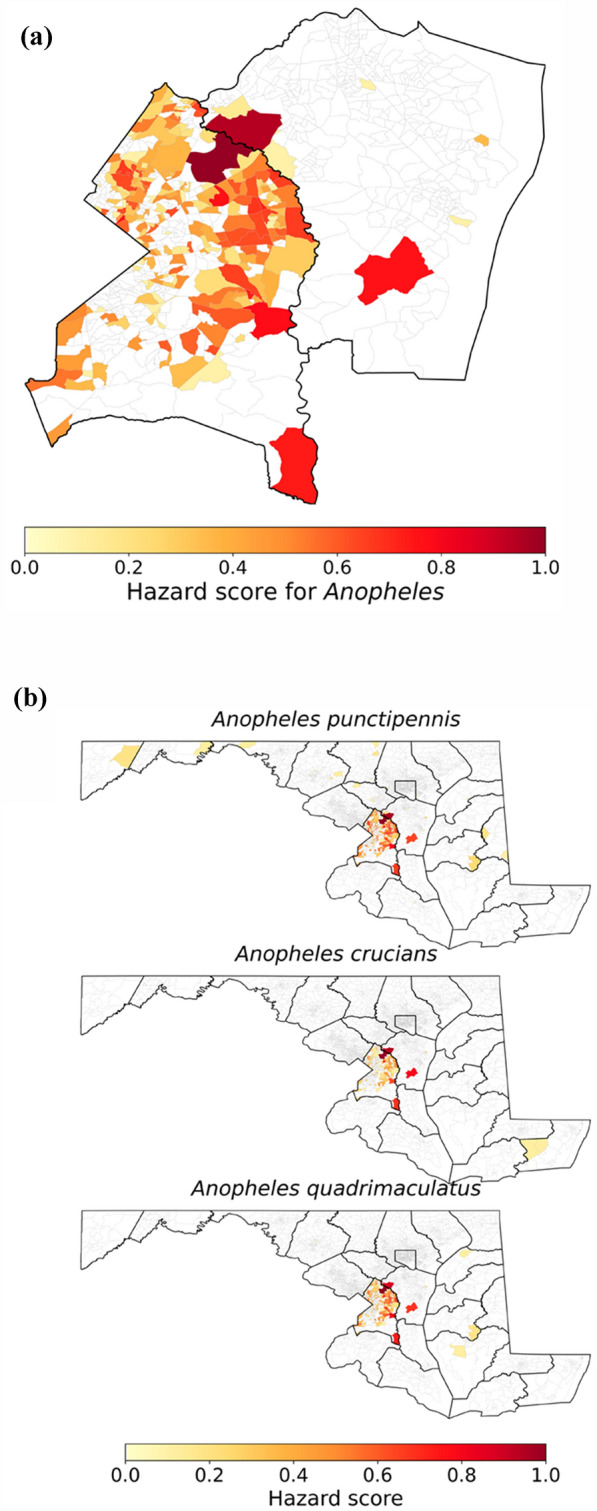
Hazard score of *Anopheles* presence at the census block group scale in Prince George’s Anne Arundel Counties (a) and across census block groups in the US for the three *Anopheles* species analysed

At the CBG scale, approximately 41% of block groups in Prince George’s County and only about 2% in Anne Arundel County have hazard scores greater than zero, indicating a wider spatial extent of reported mosquito presence in Prince George’s compared to other Maryland counties (Fig. 5a and b). The highest hazard scores are concentrated in the northeastern part of Prince George’s County and the northwestern part of Anne Arundel County.

Across the three main *Anopheles* species observed—*An. punctipennis*, *An. crucians*, and *An. quadrimaculatus*—the spatial distribution of hazard scores is fairly consistent (Fig. 5b). All three species show concentration of high hazard scores in the same northeastern Prince George’s and northwestern Anne Arundel hotspots. This consistency likely reflects shared ecological preferences among these species, including similar breeding habitat requirements and sensitivity to local climatic and land cover conditions, which potentially drive their co-occurrence at nearly the same hotspot locations.

### Correlation analysis and explainable machine learning modeling of neighborhood-scale drivers of *Anopheles* presence

**Fig. 6:**
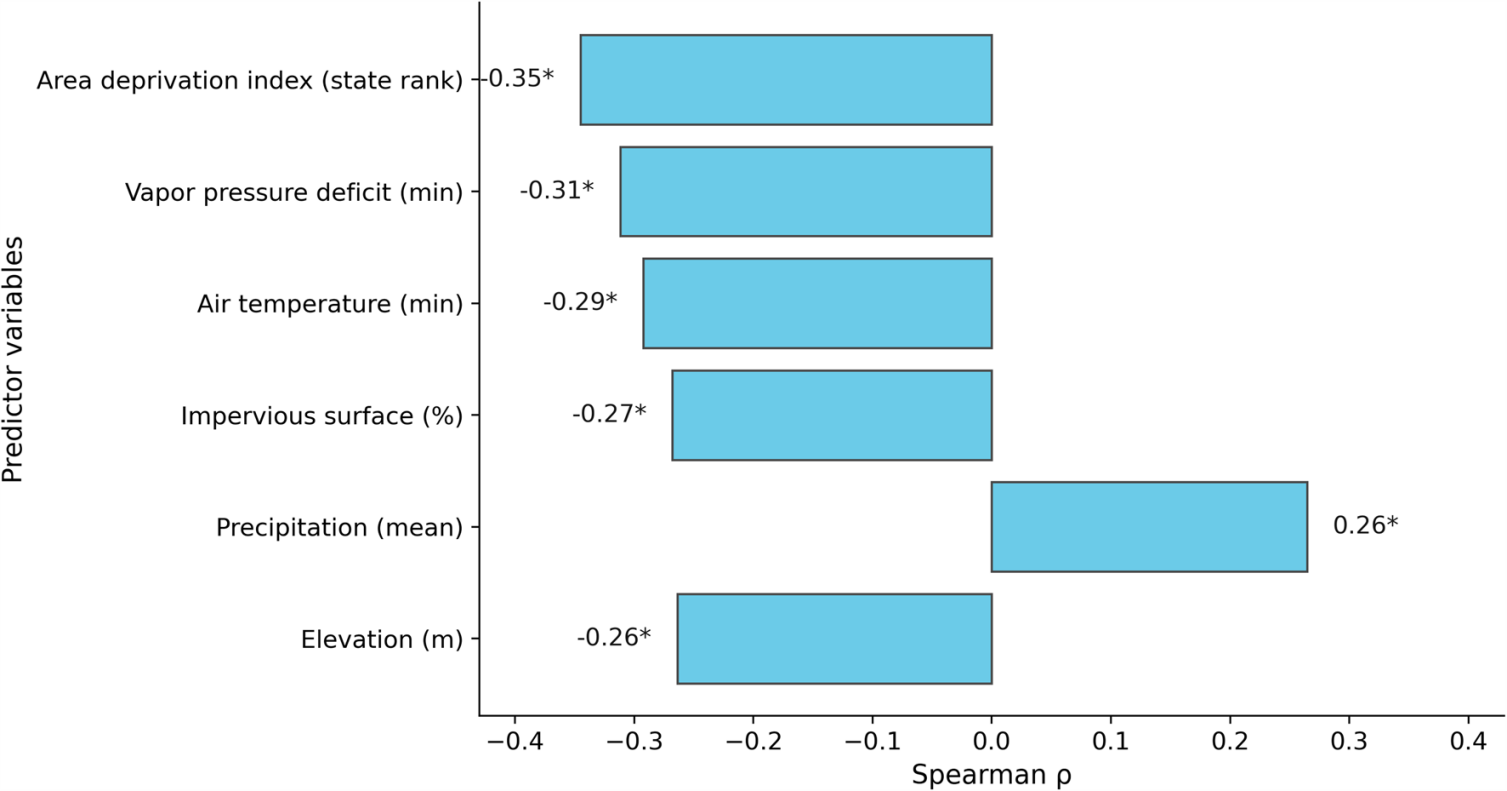
Spatial correlation at census block groups in Prince George’s County, between environmental factors, ADI and mosquito presence. Asterisk (*) shows statistical significance at 95% confidence level

Focusing on Prince George’s County, selected due to its large number of CBGs with nonzero hazard scores, Fig. 6 highlights environmental and socioeconomic variables with statistically significant correlations (|ρ| ≥ 0.25,* P* < 0.05) with *Anopheles* presence. These include the ADI, minimum vapor pressure deficit, minimum air temperature, percentage of impervious surface, mean precipitation, and elevation.

The ADI shows the strongest correlation (ρ = –0.35), indicating that unlike the non-robust county level results, at the neighborhood scale, block groups with less socioeconomic deprivation tend to have environmental conditions that favor mosquito presence. Therefore, while county-level analysis linked socioeconomic vulnerability to mosquito burden, the more reliable CBG-level analysis (robust to outliers and with relatively much higher spatial granularity: *n* = 24 counties vs 4091 CBG) within Prince George’s County revealed higher mosquito presence in less deprived neighborhoods. Unlike container-breeding mosquitoes (e.g., *Aedes* for Zika/West Nile viruses), which often proliferate in low socioeconomic status areas with abandoned properties or poor drainage [37], *Anopheles* need natural, cleaner aquatic habitats [38–40]. This pattern likely reflects fine-scale ecological drivers—such as well-maintained woody wetland habitat, low impervious surface, and humid microclimates—more prevalent in affluent residential zones.

Minimum vapor pressure deficit (ρ = –0.31) and minimum air temperature (ρ = –0.29) are also negatively correlated, suggesting that locations with lower values for these variables—potentially indicative of cooler and more humid microclimates—are associated with hotspots of *Anopheles* presence. This relationship will be further explored in the discussion section, particularly in relation to land cover and hydrological features. Impervious surface percentage (ρ = –0.27) is similarly negative, reflecting the limited breeding habitat in heavily urbanized, paved environments. This is further supported by the correlations between *Anopheles* presence and land cover fractions. Woody Wetlands Fraction achieves the highest positive correlations (ρ = 0.33) indicative of the habitat preference of the mosquito. This is followed by Herbaceous Wetlands (ρ = 0.27) and open water (ρ = 0.27). Further, from Fig. 6, mean precipitation (ρ = 0.26) is positively correlated, consistent with the role of water accumulation in supporting mosquito breeding. Finally, elevation (ρ = –0.26) is negatively correlated, suggesting that lower-lying neighborhoods—more prone to water pooling—tend to have hotspots of *Anopheles* presence.

**Fig. 7:**
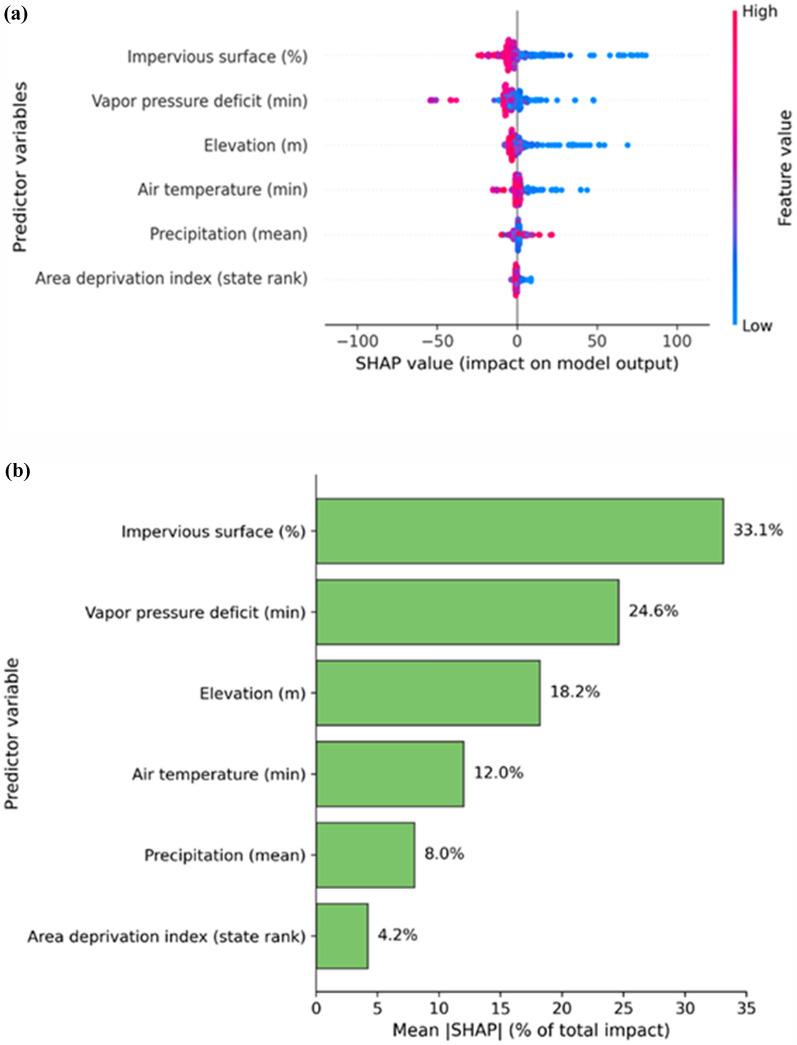
Beeswarm plot from XGBoost-SHAP modeling showing how each predictor impacts *Anopheles* mosquito presence (**a**) and the ranking of each predictor’s contribution on the model’s explanatory power (**b**)

Figure 7a and b show impact of the predictors in Fig. 6 on mosquito presence and the relative contribution of the predictors using SHAP. Impervious surface percentage emerges as the most influential predictor, accounting for 33.1% of total model impact, followed by minimum vapor pressure deficit (24.6%) and elevation (18.2%). Therefore, while the XAI model (Fig. 7) identified impervious surface as the top predictor, and correlation analysis identified woody wetlands (ρ = 0.33) as the top land cover type. Minimum air temperature ranks fourth (12.0%), consistent with its negative correlation in Fig. 7, while mean precipitation (8.0%) and ADI (4.2%) have smaller, though still meaningful, contributions.

Together, these results in Fig. 7b highlight that land surface characteristics, microclimatic moisture conditions, and socioeconomic context are notable drivers of *Anopheles* presence hotspots in the analysed county. The SHAP plots in Fig. 7a further confirm the direction of these relationships reinforcing the correlation analysis in Fig. 6: lower minimum vapor pressure deficit (more humid conditions), lower minimum air temperature (cooler microclimates), lower elevation, and lower impervious surface percentages are generally associated with higher predicted *Anopheles* hazard scores. Conversely, higher mean precipitation is linked with increased hotspots of *Anopheles* presence, consistent with its role in creating and sustaining breeding habitats. This alignment between statistical correlations and SHAP-based feature effects strengthens confidence in the stability of these predictors and their ecological plausibility.

## Discussion

At the county scale, Prince George’s and Anne Arundel Counties emerged as primary hotspots of *Anopheles* presence with high observer effort. Socioeconomic indicators—unemployment, prevalence of multi-unit housing, lack of a high school diploma, and household crowding—were positively correlated with county-level *Anopheles* presence (*P* < 0.05) using Pearson correlations. However, given the small county sample (*n* = 24), potential dominance by high-occurrence counties, and the loss of fine-scale spatial detail, these associations (which are non-significant using Spearman correlations) may reflect spatial aggregation and risk an ecological fallacy. Consistent with this concern, our CBG-level analysis within Prince George’s County showed a negative correlation between Area Deprivation Index and *Anopheles* presence (ρ = − 0.35). This scale-dependent contrast highlights the need for cautious interpretation, considering sampling bias, observer effort, and aggregation effects.

The CBG-scale analysis revealed heterogeneity of recorded presence within counties. Prince George’s County stood out, with approximately 41% of its block groups showing nonzero hazard scores. This spatial pattern indicates the importance of fine-resolution analyses for detecting localized hotspots that may be masked in coarser-scale assessments.

A key insight from our analysis is the role of land cover and microclimatic moisture conditions. At the CBG, low impervious surface percentage (33.1% of total SHAP) and minimum vapor pressure deficit (VPDmin, 24.6% of total impact) emerged as important predictors of *Anopheles* presence hotspots. This was followed by elevation, and minimum air temperature.

The strong positive correlation between VPDmin and minimum air temperature (ρ = 0.98) suggests that lower nighttime temperatures during warm months often coincide with more humid microenvironments. This is further supported by Table S1, which shows strong negative correlations between VPDmin and forested or agricultural land use types: land covers that typically provide shade, reduce local temperatures, and help retain atmospheric moisture. In contrast, the positive correlation between VPDmin and impervious surface fractions in developed areas supports the idea that urban heat retention and reduced vegetation contribute to higher VPD and atmospheric drying, consistent with the urban heat island effect (Table S1).

As shown in Table S1, these moisture-rich microclimates—often associated with shaded wetlands, floodplains, or vegetated lowlands—provide the stable humidity and breeding habitats required for *Anopheles* survival and reproduction [41]. For our area of assessment, woody wetland, typically associated with saturated soils that provide breeding grounds for larvae, relatively lower temperatures, dense vegetation, and high humidity emerged as potentially the most preferred habitat for *Anopheles* mosquitoes. Moreover, a laboratory study by Kessler and Guerin [42] have demonstrated that *Anopheles* adults preferentially seek cooler, more humid microhabitats when deprived of water or sugar, using thermohygroreceptor cells to detect favorable conditions. In these settings, lower saturation deficits reduce metabolic stress and improve survival prospects. This behavioral adaptation is consistent with our finding at the CGB-scale that lower minimum temperatures, which often signal more humid microenvironments, are associated with higher hotspots of *Anopheles* presence in Prince George’s County. The concordance between our field-based analysis and controlled laboratory experiments provides strong biological plausibility for the observed relationship between microclimatic humidity and *Anopheles* hazard.

These findings help reconcile the seemingly counterintuitive negative correlation between hazard scores and minimum temperatures. While high daytime temperatures during summer (June to August, Fig. 4) promote rapid mosquito development, cooler nighttime minima in certain landscapes may signal the presence of nearby water bodies, wetlands, or dense vegetation that retain humidity, creating microhabitats favorable for *Anopheles* survival and reproduction [41]. Such moisture-rich environments buffer mosquitoes from desiccation stress and can extend adult lifespan [42]. Our SHAP analysis supports this interpretation, revealing that both humidity proxies (VPDmin) and habitat features (elevation, impervious surface cover) exert strong influence on hotspots of *Anopheles* presence at neighborhood scales, highlighting the interplay between microclimate and landscape structure in shaping local vector ecology.

As earlier mentioned, seasonal patterns emerged clearly in the temporal analysis. At the hotspot counties, mosquito presence peaked between July and August, following an increase from May and declining sharply from October. This pattern reflects the combined influence of seasonal warmth—accelerating mosquito development [16]—and localized humidity [42]—extending survival and breeding potential. Together, these seasonal and microclimatic drivers contribute to explaining why hotspots of *Anopheles* presence is concentrated in specific sub-county zones despite relatively uniform summer warmth across the broader region.

Finally, our study highlights the novelty and importance of fine spatial-scale analysis. By working at the CBG level, we were able to identify micro-scale patterns—such as the link between cooler nighttime temperatures, higher humidity, and *Anopheles* presence —that would be obscured in coarser analyses. This granularity is particularly valuable for targeted surveillance and control strategies, allowing public health agencies to focus resources on the most at-risk neighborhoods (Figs. S1 and S2) rather than entire counties.

Limitations of our study include the reliance on GBIF presence-only occurrence data, which are subject to non-random sampling bias. Identified hotspots may partly reflect areas of high sampling effort rather than true mosquito abundance, while zero-record counties may simply be undersampled. The strong county-level correlations observed (Fig. 3) are also sensitive to spatial aggregation and outlier influence, whereas the finer CBG-level analysis captures more realistic ecological patterns of the species. The sharp post-2019 declines (Fig. 4) may reflect reduced surveillance or reporting during the COVID-19 pandemic, rather than ecological change. Additionally, although environmental predictors were derived from high-resolution datasets, they may not fully represent small-scale breeding habitats such as artificial containers, irrigation systems, or microclimatic variations. Finally, we acknowledge temporal mismatch among data sources (Table 1); our approach intentionally uses the most recent, high-quality covariate snapshots as time-stable environmental baselines for spatial modeling. Despite these limitations, the combined use of correlation analysis, SHAP-based model interpretation, and multi-scale spatial analysis offers a robust and interpretable framework for understanding *Anopheles* dynamics in a temperate setting.

Building on these limitations, several methodological refinements could strengthen future work. First, observer effort could be modeled more explicitly by incorporating effort proxies—such as observer-days, program-based versus opportunistic records, or structured surveillance subsamples—as covariates or offsets to better separate true ecological signal from sampling intensity. Second, although our use of long-term climatological normals provides a stable environmental baseline for defining typical conditions across Maryland, future analyses could complement this approach with temporally resolved covariates or decadal subsets to capture interannual variability. Third, at the county scale, multivariate frameworks (e.g., penalized regression or hierarchical models) could account for confounders such as population density or urbanization, surpassing the reported limitations of pairwise correlations in small samples. Finally, alternative distributional assumptions for count data—such as Poisson, negative binomial, or zero-inflated models—could be tested alongside gradient-boosted regression to benchmark predictive performance and assess residual spatial autocorrelation. Collectively, these enhancements would allow future versions of this framework to more fully disentangle ecological patterns from sampling biases and to advance hazard estimation for *Anopheles* mosquitoes in temperate environments.

## Conclusions

This study combined long-term GBIF occurrence records with explainable machine learning and correlation analysis to examine the environmental and socioeconomic factors influencing the presence of *Anopheles* mosquitoes (*An. punctipennis, An. crucians, and An. quadrimaculatus*) across Maryland at both county and Census Block Group (CBG) scales. The analysis identified Prince George’s County as the primary hotspot of *Anopheles* presence in the state, supported by sufficient observer effort to allow robust spatial inference. Seasonal patterns further indicated that warm summer temperatures provide optimal climatic conditions for *Anopheles* proliferation in identified hazard hotspots. At the CBG scale, favorable breeding habitats—including woody wetlands, herbaceous wetlands, and open water—together with low minimum vapor pressure deficit emerged as the most influential environmental factors shaping mosquito suitability. These were followed by lower elevation, lower minimum temperatures, and higher precipitation, which collectively contribute to the persistence of humid, water-rich microenvironments conducive to mosquito development. Socioeconomic analysis using the Area Deprivation Index suggested that *Anopheles* mosquitoes may be more prevalent in CBGs with lower levels of social deprivation, contrasting with container-breeding mosquitoes such as *Aedes aegypti*, which are often associated with higher deprivation and urban decay. Overall, the findings demonstrate the value of integrating occurrence data with interpretable machine learning to resolve multi-scale patterns of *Anopheles* presence, providing a foundation for spatially targeted vector surveillance and public health preparedness. Future work will extend this framework by using the most informative feature combinations identified here to train predictive models capable of estimating *Anopheles* hazard across CBGs throughout the United States.

## Supplementary Information

Supplementary file 1.

## Data Availability

GBIF species occurrence data used in this study are publicly available at https://www.gbif.org, where users can access metadata and download datasets. Additional processed datasets generated during the analysis are available from 10.5281/zenodo.17833283

## Abbreviations

ADI: Area deprivation index
AI: Artificial Intelligence
ATSDR: Agency for toxic substances and disease registry
CBG: Census block group
CDC: Centers for disease control and prevention
DEM: Digital elevation model
GBIF: Global biodiversity information facility
ML: Machine learning
MRLC: Multi-resolution land characteristics consortium
NHD: National hydrography dataset
NLCD: National land cover database
PRISM: Parameter-elevation regressions on independent slopes model
SHAP: SHapley Additive exPlanations
SVI: Social vulnerability index
USGS: United States geological survey
VPD: Vapor pressure deficit
XAI: Explainable artificial intelligence
XGBoost: Extreme gradient boosting

## References

[1] LaRocque RL, Ryan ET. Personal actions to minimize mosquito-borne illnesses, including Zika virus. Ann Intern Med. 2016;165(8):589–90. 27399646 10.7326/M16-1397

[2] Dambach P. The use of aquatic predators for larval control of mosquito disease vectors: opportunities and limitations. Biol Control. 2020;150:104357.

[3] Coşgun Y, Bayrakdar F, Akiner MM, Gürer Giray B, Demirci B, Bedir H, et al. Investigation of the presence of Zika, Dengue, Chikungunya, and West Nile virus in *Aedes* type mosquitoes in the Eastern Black Sea area of Turkey. Turk Bul Hifz Tecr Biol. 2023;80(1):101–8.

[4] Namango IH, Moore SJ, Marshall C, Saddler A, Kaftan D, Tenywa FC. A matter of timing: biting by malaria-infected *Anopheles* mosquitoes and the use of interventions during the night in rural south-eastern Tanzania. PLOS Glob Public Health. 2024;4(12):e0003864. 39739884 10.1371/journal.pgph.0003864 PMC11687804

[5] Penhollow TM, Torres L. Impact of mosquito-borne diseases on global public health. Int Phys Med Rehabilit J. 2021;6(1):19–20.

[6] Wang GH, Gamez S, Raban RR, Marshall JM, Alphey L, Li M, et al. Combating mosquito-borne diseases using genetic control technologies. Nat Commun. 2021;12(1):4388. 34282149 10.1038/s41467-021-24654-z PMC8290041

[7] Näslund J, Ahlm C, Islam K, Evander M, Bucht G, Lwande OW. Emerging mosquito-borne viruses linked to *Aedes aegypti* and *Aedes albopictus*: global status and preventive strategies. Vector Borne Zoonotic Dis. 2021;21(10):731–46. 34424778 10.1089/vbz.2020.2762

[8] Bera B. Mosquito ecology and disease transmission: implications for global health and vector control. Uttar Pradesh J Zool. 2025;46(8):64–72.

[9] Caraballo H, King K. Emergency department management of mosquito-borne illness: malaria, dengue, and West Nile virus. Emerg Med Pract. 2014;16(5):1–23. 25207355

[10] Lindsey NP, Lehman JA, Staples JE, Fischer M. West Nile virus and other arboviral diseases—United States, 2013. Am J Transplant. 2014;14(10):2422–6. PMC5779373 24941331

[11] Dieme C, Rotureau B, Mitri C. Microbial pre-exposure and vectorial competence of *Anopheles* mosquitoes. Front Cell Infect Microbiol. 2017;7:508. 29376030 10.3389/fcimb.2017.00508 PMC5770632

[12] Shone SM, Ferrao PN, Lesser CR, Norris DE, Glass GE. Analysis of mosquito vector species abundances in Maryland using geographic information systems. Ann N Y Acad Sci. 2001;951(1):364–8. 11797801 10.1111/j.1749-6632.2001.tb02720.x

[13] LaDeau SL, Leisnham PT, Biehler D, Bodner D. Higher mosquito production in low-income neighborhoods of Baltimore and Washington, DC: understanding ecological drivers and mosquito-borne disease risk in temperate cities. Int J Environ Res Public Health. 2013;10(4):1505–26. 23583963 10.3390/ijerph10041505 PMC3709331

[14] Little E, Biehler D, Leisnham PT, Jordan R, Wilson S, LaDeau SL. Socio-ecological mechanisms supporting high densities of *Aedes albopictus* (Diptera: Culicidae) in Baltimore, MD. J Med Entomol. 2017;54(5):1183–92. 28605549 10.1093/jme/tjx103 PMC5850657

[15] Kutz FW, Wade TG, Pagac BB. A geospatial study of the potential of two exotic species of mosquitoes to impact the epidemiology of West Nile virus in Maryland. J Am Mosq Control Assoc. 2003;19(3):190–8. 14524539

[16] Ibebuchi CC, Abu IO, Onwah SS. Environmental factors contributing to southern house mosquito presence in Clark County, Nevada, using machine learning. Environ Res Commun. 2025;7(6):061005.

[17] Shone SM, Glass GE, Norris DE. Targeted trapping of mosquito vectors in the Chesapeake Bay area of Maryland. J Med Entomol. 2006;43(2):151–8. 16619593 10.1603/0022-2585(2006)043[0151:ttomvi]2.0.co;2 PMC4152323

[18] Ngape D, Steele CH, McDermott EG. A comparison of BG Sentinel and CDC trap attractants for mosquito surveillance in urban and suburban areas of Montgomery and Prince George’s Counties, Maryland, USA. J Vector Ecol. 2021;46(2):186–99. 35230023 10.52707/1081-1710-46.2.186

[19] Rothman SE, Jones JA, LaDeau SL, Leisnham PT. Higher West Nile virus infection in *Aedes albopictus* (Diptera: Culicidae) and *Culex* (Diptera: Culicidae) mosquitoes from lower income neighborhoods in urban Baltimore, MD. J Med Entomol. 2021;58(3):1424–8. 33257956 10.1093/jme/tjaa262

[20] Ewing DA, Cobbold CA, Purse BV, Nunn MA, White SM. Modelling the effect of temperature on the seasonal population dynamics of temperate mosquitoes. J Theor Biol. 2016;400:65–79. 27084359 10.1016/j.jtbi.2016.04.008

[21] Montgomery MJ, Harwood JF, Yougang AP, Wilson-Bahun TA, Tedjou AN, Keumeni CR, et al. The effects of urbanization, temperature, and rainfall on *Aedes aegypti* and *Aedes albopictus* mosquito abundance across a broad latitudinal gradient in Central Africa. Parasit Vectors. 2025;18(1):135. 40189559 10.1186/s13071-025-06764-5 PMC11972486

[22] CDC. Notes from the Field: Locally Acquired Mosquito-Transmitted (Autochthonous) *Plasmodium falciparum* Malaria — National Capital Region, Maryland, August 2023. https://www.cdc.gov/mmwr/volumes/72/wr/mm7241a3.html. Accessed 9 Aug 2025.

[23] Maryland Department of Health. Maryland Department of Health announces positive case of locally acquired malaria. https://health.maryland.gov/newsroom/Pages/Maryland-Department-of-Health-announces-positive-case-of-locally-acquired-malaria.aspx. Accessed 10 July 2025.

[24] Little EA, Hutchinson M, Price KJ, Marini A, Shepard JJ, Molaei G. Spatiotemporal distribution, abundance, and host interactions of two invasive vectors of arboviruses, *Aedes albopictus* and *Aedes japonicus*, in Pennsylvania, USA. Parasit Vectors. 2022;15(1):36. 35073977 10.1186/s13071-022-05151-8 PMC8785538

[25] Lee CH, Leonard M, Smith RC. Abundance, distribution, and dynamics of *Anopheles* species (Diptera: Culicidae) in Iowa, United States. J Med Entomol. 2024;61(6):1391–8. 39158078 10.1093/jme/tjae098

[26] Borton D. Preventing malaria spread in the US. Nurs. 2024;54(3):21–8. 10.1097/01.NURSE.0001006264.42321.ff 38386446

[27] Courtney AP. Locally acquired (autochthonous) mosquito-transmitted *Plasmodium vivax* malaria—Saline County, Arkansas, September 2023. MMWR Morb Mortal Wkly Rep. 2024. 10.15585/mmwr.mm7342a2. 39446670 10.15585/mmwr.mm7342a2 PMC11500839

[28] GBIF.org. GBIF Occurrence Download. 2025. https://gbif.org/

[29] Maryland Department of Agriculture. Mosquito Control. Retrieved August 11, 2025, from https://mda.maryland.gov/plants-pests/pages/mosquito_control.aspx. Accessed 10 July 2025.

[30] Nanfack Minkeu F, Vernick KD. A systematic review of the natural virome of anopheles mosquitoes. Viruses. 2018;10(5):222. 29695682 10.3390/v10050222 PMC5977215

[31] Kind AJH. Area Deprivation Index (ADI) (Version 4.0.1) . University of Wisconsin School of Medicine and Public HealthNeighborhood Atlas. 2023. https://www.neighborhoodatlas.medicine.wisc.edu/download

[32] Centers for Disease Control and Prevention/Agency for Toxic Substances and Disease Registry/Geospatial Research, Analysis, and Services Program (CDC/ATSDR). (2022). CDC/ATSDR Social Vulnerability Index 2022 Database: United States. https://www.atsdr.cdc.gov/place-health/php/svi/svi-data-documentation-download.html

[33] PRISM Climate Group (2020) 30-year Normals: https://prism.oregonstate.edu/normals/

[34] Multi-Resolution Land Characteristics Consortium (MRLC 2024) NLCD: . https://www.mrlc.gov/data

[35] Wegener C, Ibebuchi CC. Application of xgboost in disentangling the fingerprints of global warming and decadal climate modes on seasonal precipitation trends in ohio. Int J Climatol. 2025;45(8):e8829.

[36] Ibebuchi CC. Uncertainty in machine learning feature importance for climate science: a comparative analysis of shap, pdp, and gain-based methods. Theor Appl Climatol. 2025;156(9):1–14.

[37] Yitbarek S, Chen K, Celestin M, McCary M. Urban mosquito distributions are modulated by socioeconomic status and environmental traits in the USA. Ecol Appl. 2023;33(5):e2869. 37140135 10.1002/eap.2869

[38] Khan GZ, Khan IA, Khan I, Inayatullah M. Outdoor breeding of mosquito species and its potential epidemiological implications in Khyber Pakhtunkhwa. Pak J Agric Res. 2014;27:4.

[39] Young GB, Golladay S, Covich A, Blackmore M. Stable isotope analysis of larval mosquito diets in agricultural wetlands in the coastal plain of Georgia, USA. J Vector Ecol. 2014;39(2):288–97. 25424257 10.1111/jvec.12103

[40] Onchuru TO, Ajamma YU, Burugu M, Kaltenpoth M, Masiga D, Villinger J. Chemical parameters and bacterial communities associated with larval habitats of Anopheles, Culex and Aedes mosquitoes (Diptera: Culicidae) in western Kenya. Int J Trop Insect Sci. 2016;36(3):146–60.

[41] Yamana TK, Eltahir EA. Incorporating the effects of humidity in a mechanistic model of *Anopheles gambiae* mosquito population dynamics in the Sahel region of Africa. Parasit Vectors. 2013;6(1):235. 23938022 10.1186/1756-3305-6-235 PMC3750695

[42] Kessler S, Guerin PM. Responses of *Anopheles gambiae*, *Anopheles stephensi*, *Aedes aegypti*, and *Culex pipiens* mosquitoes (Diptera: Culicidae) to cool and humid refugium conditions. J Vector Ecol. 2008;33(1):145–9. 18697317 10.3376/1081-1710(2008)33[145:roagas]2.0.co;2

