# Supplementary material for: Microclimates, land cover, and socioeconomic vulnerability shape Anopheles hotspots in Maryland, USA
Source: Infect Dis Poverty. 2026 Jan 20;15:12. doi: 10.1186/s40249-025-01407-4 (PMC12817407; doi:10.1186/s40249-025-01407-4)
Supplement: Supplementary file 1 [file 40249_2025_1407_MOESM1_ESM.docx]

**Microclimates, Land Cover, and Socioeconomic Vulnerability Shape *Anopheles* Hotspots in Maryland**

Supplementary file

**Table S1:** Correlation between VPDMin at CBGs in Maryland and Land cover types (2024) from NLCD.

| **Land Cover Type** | **Spearman rho** | **Interpretation** |
| --- | --- | --- |
| **Pasture/Hay** | −0.418 | High moisture retention; open canopy allows dew formation |
| **Deciduous Forest** | −0.397 | Dense canopy and transpiration maintain humid microclimate |
| **Cultivated Crops** | −0.370 | Irrigation and canopy cover contribute to lower VPD |
| **Mixed Forest** | −0.336 | Diverse canopy structure enhances moisture buffering |
| **Evergreen Forest** | −0.293 | Year-round foliage supports consistent transpiration |
| **Grass/Herbaceous** | −0.273 | Ground cover slows evaporation, retains surface moisture |
| **Shrub/Scrub** | −0.272 | Similar to grasslands; moderate evapotranspiration |
| **Woody Wetlands** | −0.164 | Saturated soils and vegetation maintain high humidity |
| **Open Water** | −0.138 | Evaporation contributes to local humidity, but less canopy buffering |
| **Deloped Medium** | +0.301 | Impervious surfaces and heat retention increase VPD |
| **Developed High** | +0.291 | Urban heat island effect drives atmospheric drying |
